# Challenges and Barriers to HIV Care for Mexican Born Men Living in Chicago

**DOI:** 10.1007/s10903-021-01158-y

**Published:** 2021-02-22

**Authors:** Patricia Aguado, Susan Ryerson-Espino, Pamela Vergara-Rodriguez

**Affiliations:** 1 Department of Social Work, Northeastern Illinois University, 5500 North St. Louis Avenue, Lech Welesa Hall, Chicago, IL 3043 USA; 2 The Ruth M. Rothstein CORE Center, Hektoen Institute of Medicine, Chicago, IL USA; 3 Cook County Bureau of Health Services, The Ruth M. Rothstein CORE Center, Chicago, IL USA

**Keywords:** Mexican, Latino/x, Mental health, Barriers, People living with HIV, Men

## Abstract

*Background*: Latinx men are disproportionately impacted by HIV. Research often looks at Latinx people as a heterogeneous population. This paper describes baseline characteristics and barriers to HIV care among Mexican born men enrolled in an HIV care engagement intervention at a public health clinic in Chicago. *Methods*: Survey and medical chart data were collected. *Results*: 66 Mexican born men enrolled in the project. Over half (60%) were newly diagnosed; 40% were reengaging in care or establishing care for the first time. Participants reported significant pre and postmigration concerns including poverty, social stigma, late entry to care, and concurrent health concerns, including 47% screening positive for depression. Barriers to care and mental health concerns were significantly related to Stage 3 HIV. *Discussion*: More prevention and intervention research is needed to ameliorate the negative socioeconomic and health ramifications of immigration and bolster mental and sexual health, reduce HIV transmission, and increase testing, linkage and care retention.

**Supplementary Information:**

The online version contains supplementary material available at 10.1007/s10903-021-01158-y.

## Background

Next to Black Americans, Latinxs are the group most impacted by HIV in the United States; they have lower levels of HIV care retention, lower HIV viral suppression rates and experience HIV related deaths two times higher than do non-Latinx Whites [1]. Among Latinxs, between 2010 and 2016, new HIV diagnoses increased by 10%, retention in HIV medical care increased for all other racial/ethnic subgroups but remained the same for Latinxs, and while Latinxs make up 18% of the total population, they accounted for 28% of new infections nationally [1, 2].

In 2017, Illinois was seventh among the states reporting the highest number of HIV diagnoses [3]. The city of Chicago carries the burden of HIV in Illinois and is home to roughly 24,000 people living with HIV/AIDS (PLWHA) [4]. Men in Chicago accounted for 5.2 times as many new HIV diagnoses when compared to women, men who have sex with men (MSM) accounted for the majority (72.2%) of new HIV diagnoses, and men were significantly more likely to present with late-stage HIV diagnosis (defined as having AIDS at the time of initial HIV diagnosis or being diagnosed with AIDS within 12 months of initial diagnosis) [4, 5]. Latinxs accounted for 22% of new HIV diagnoses and made up 24% of those presenting with late-stage HIV diagnoses. Given the incidence and prevalence of HIV among the Latinx community nationally and locally in Chicago, it is important to have a better understanding of the driving factors of HIV/AIDS and linkage and retention to care among Latinx subgroups, like Mexican immigrant men.

The prevalence of depression, anxiety and substance use have long been recognized as influential in the linkage and retention and overall quality of life of people living with HIV/AIDS (PLWHA), including Latinxs [6–8]. Foreign-born Latinx PLWHA often bear the added hardships of migration stress and trauma [9, 10]. Mexican-born individuals living in the US are more likely to endure economic challenges, lack health insurance and immigration documentation, experience language and other barriers to care as well as inferior HIV-related outcomes [11–13]. Moreover, this group is more likely to have experienced trauma before and during migration to the United States. Sociocultural factors such as lack of HIV knowledge, health care practices carried over from Mexico, work schedules, Machismo, stigma related to homosexuality, and internalized homophobia also present challenges to the identification and retention of individuals living with HIV [12, 13]. HIV alone may not be the major challenge Mexican immigrants experience, but rather the interplay of mental health issues, sociocultural and structural barriers contribute collectively to health disparities affecting Latinx PLWHA [11–14]

Much of what is known about HIV among Latinx communities is broad in scope; when researching immigrants, Latinxs are often grouped together without focus on any specific subgroup. This paper’s aim is to add to the literature by describing the characteristics and barriers to HIV care and retention for Mexican immigrant men living in Chicago.

### Conceptual Framework

Ecological Systems Theory informed the conceptual framework for this study. Specifically, we aimed to explore multi-level barriers to engagement in HIV care work including interpersonal, community and structural barriers [15].

#### Description

In September 2013, the Cook County Health and Hospital Systems (CCHHS), Ruth M. Rothstein CORE Center was one of ten sites nationally funded by the Health Resources and Services Administration (HRSA), Special Project of National Significance (SPNS) to develop a transnationally-informed intervention whose focus was to better understand and address HIV care continuum barriers for a Latino subpopulation. *Proyecto Promover* was born out of this opportunity and aimed to understand multi-level barriers to care and increase the identification, engagement, and retention in care of Mexican-identified individuals living with HIV in Chicago.

The current study highlights lessons learned from baseline data which were used as an early step to individualizing the patient-centered navigation that formed the primary clinic-based intervention component of *Proyecto Promover*. Baseline data also provide an important highlight of barriers facing low income Mexican identified individuals seeking care in Chicago and may be informative for the development of other prevention and care engagement interventions within Chicago and in other cities with similar populations.

## Methods

### Eligible Participants

*Proyecto Promover* was a prospective study of Mexican identified individuals of all gender identities living with HIV in Chicago and the surrounding areas. Participants were enrolled between January 2015 and October 2017 from an HIV primary care clinic in the city of Chicago. Eligibility criteria included: self-identifying as Mexican; aged 18 years or older; and, newly diagnosed with HIV (within the past six months) or not optimally engaged in care (diagnosed more than six months ago and no HIV primary care visits in the last six months). Participants did not have to be literate but had to be able to speak English or Spanish and provide verbal consent. For the purpose of this study we limited participation to Mexican-identified, born in Mexico, self-identified men.

### Data Collection

Baseline data were gathered via two methods. A computer-assisted self-interview (ACASI) developed by the national evaluation and technical assistance center (ETAC), at the University of California at San Francisco (UCSF) and local structured interviews between clinical navigators and patients. The current study also involved baseline medical chart data. All survey instruments and interview protocols were either published Spanish versions or locally translated from English to Spanish, then back translated from Spanish to English by a certified local Mexican-identified translator. This study was approved by the Institutional Review Boards at the CCHHS and the UCSF ETAC.

### Measures

The multisite baseline quantitative survey was administered at the time of consent or soon thereafter most typically at the time of a first clinical linkage appointment. This survey elicited demographics including age, preferred gender, education, marital status, income, insurance, sexual attraction, and sexual orientation. This survey also elicited transnational experiences including birthplace, travel between Mexico and the United States, age of first migration, English and Spanish language proficiencies, and remittances. Local sites were able to incorporate a few measures into the ACASI; local *Proyecto Promover* items explored immigrant stressors, family separations, and exposure to domestic violence. *Proyecto Promover’s* intervention and evaluation team members reviewed the Hispanic Stress Inventory [16] and identified items for inclusion and adaptation. Ultimately six items explored premigration economic stressors, four items explored premigration social stigma, five explored journey trauma, and two items explored postmigration housing (appended). Participants were asked to identify using a checklist any family and significant friend separations of one year or more, as a result of living in the USA. Participants were also asked to report current and past exposure to intimate partner violence adapted from a Women of Color HRSA-SPNS initiative [17].

In addition to the ACASI, participants and the clinical navigators met to discuss the intervention and explore barriers to care. These interviews occurred within the first 2 months of their engagement in care and helped individualize the patient-centered navigation intervention. This local structured interview included the Primary Care PTSD Screen [18], the Center for Epidemiologic Studies Depression Scale (CES-D) [19], the CAGE questionnaire [20], and a barriers inventory, also adapted from a Women of Color HRSA SPNS initiative [17] that explored barriers to care including awareness of resources, coping with HIV and treatment, and HIV stigma. All scales used in this study were found to have acceptable internal reliability as measured by Cronbach’s alpha as reported in Tables 2 and 3, along with computation of scale scores.

Medical chart data reviewed included date of diagnosis, care continuum status (newly diagnosed or not optimally engaged in care) and clinical characteristics at linkage to clinic: viral load, CD4, presence of opportunistic infections (OIs), concurrent syphilis, and if participant was referred to intervention from an acute care setting.

### Data Analysis

Data were merged and analyzed using IBM SPSS Statistics 26. Descriptive statistics (means, medians, standard deviations, ranges, averages, and frequency distributions) were used to understand baseline characteristics. Measures of association, test of normality and independent samples t test were used to explore relationships between baseline characteristics, specifically demographic, transnational and clinical variables.

Months since diagnosis was computed using dates of diagnosis and consent and helped validate care continuum status. Age of migration and diagnosis were compared to determine likely country of transmission (U.S. if after the date of migration). Suppression was identified for all with viral loads < 200 mL. Stage 3 HIV was determined by reviewing the length of time since diagnosis, CD4 counts, and OIs documented in the medical record. Specifically, Stage 3 was identified when diagnosis < 3 months and CD4 counts < 350 or there was evidence in the medical record of OIs.

## Results

### Baseline Demographic Characteristics

Between January 2015 and July 2017, 66 Mexican identified and born men enrolled in Proyecto Promover and completed the baseline survey. Participants were on average 38 years old; only 35% completed high school or higher in education. Of those reporting income, three quarters (78%) reported income below $11,491 (single adult federal poverty line in 2013 when study was designed) and were largely uninsured (62%). The majority (74%) appeared to be stably housed within the six months prior to baseline. The majority of participants identified as single (60%); 70% of the men endorsed sexual attraction to men (20% some to 50% mostly/only attracted to males). Relative to sexual orientation, 24% did not make a selection and an additional 6% endorsed “other” indicating that terms used may not be recognized/accepted; 36% identified as gay, 17% endorsed “straight”, and 15% as “bi” Table 1.

**Table 1:** Demographics

Variables	Descriptive statistics (n = 66)
Current age	m = 38.2 (sd = 8.4), range 22–63
Preferred Gender Cis Men	100% (66)
Education I haven’t gone to school Grade 4 or less Primary and two years of secondary (5th-8th) Third year of secondary and two years of prep (9–11) Grade 12 or GED (or prep exam) Some college, associates or technical degree Bachelors degree	1.5% (1) 9.1% (6) 33.3% (22) 21.2% (14) 24.2% (16) 9.1% (6) 1.5% (1)
Marital status single In relationship – living together In relationship – not living together Married	60.6% (40) 16.7% (11) 13.6% (9) 9.1% (6)
Income in last 12 months < $600 $600–2900 $3000–5999 $6000–11,490 $11,491–15,282 $15,283–35,999 Don’t know	21.2% (14) 19.7% (13) 15.2% (10) 10.6% (7) 15.2% (10) 3% (2) 15.2% (10)
Homelessness/Housing instability in last 6 months never Rarely or only a few times Sometimes Often	74.2% (49) 13.6% (9) 10.6% (7) 1.5% (1)
Insurance No Yes Don’t know	62.1% (41) 33.3% (22) 4.5% (3)
Sexual attraction only females Mostly males Only males Equally males & females Mostly females	30.3% (20) 25.8% (17) 24.2% (16) 12.1% (8) 7.6% (5)
Sexual orientation gay No selection Straight Bisexual Multi (gay and bisexual) Other	36.4 (24) 24.2% (16) 16.7 (24) 15.2% (10) 1.5% (1) 6.1% (4)

### Transnational Experiences

All study participants (100%) were immigrants from Mexico arriving in the U.S. on average 17 years ago at an average age of 21 years. The majority of participants (73%) reported never traveling to Mexico; 97% reported family separations, most commonly from parents (77%); 80% reported occasional to regular remittances to family back in Mexico; English language proficiency varied; one quarter (24%) reported speaking English well to very well; the majority (97%) reported speaking Spanish well to very well. The vast majority (93%) reported at least some economic concerns in Mexico, 65% reported at least some social stigma in Mexico, and 47% reported being exposed to at least one trauma during journey(ies) to the United States. Over half (62%) reported at least some exposure to poor housing conditions in the states Table 2.

**Table 2:** Transnational experiences

Variable	Descriptive statistics (n = 66)
Birthplace Mexico	100% (66)
Age of first migration (n = 77)	M = 21.1 yrs (SD = 6.2), Range 4–39
Travel to Mexico Never Rarely Sometimes Often	72.7% (48) 18.2% (12) 7.6% (5) 1.5% (1)
Separations any family/close friend separation One or more parents Extended family Close friend(s) Immediate family (spouse and or children)	97% (64) 77.3% (51) 57.6% (38) 37.9% (25) 7.6% (5)
Remittances occasionally Regularly No	56.1% (37) 24.2% (16) 19.7% (13)
English language proficiency (6 items, summed and averaged, Cronbach’s α = .97)	M = 2.2 (SD = .8), Range 1–4 (26.2% Well-Very Well)
Spanish language proficiency (6 items, summed and averaged, Cronbach’s α = .94)	M = 3.8 (SD.4),Range 2.7–4.0 (94% Well-Very Well)
Premigration economic stressors (6 items, summed and averaged, Cronbach’s α = .76; 1 “nunca”—4 “todo el tiempo”)	M = 2.1 (SD = .7), Range 1–4
Never	7.6% (5)
At least some economic concerns	92.4% (61)
Premigration social stigma (4 items, summed and averaged, Cronbach’s α = .89; 1 “nunca”—4 “todo el tiempo”)	M = 1.7 (SD = .9), Range 1–4
Never	34.8% (23)
At least some social stigma	65.2% (43)
Journey trauma (5 items, summed and averaged, Cronbach’s α = .76; 1 “nunca”—4 “todo el tiempo”) Never Exposed to at least one trauma	M = 1.3 (SD = .5), Range 1–3.2 53% (35) 46.7% (31)
US housing conditions (2 items, summed and averaged, Cronbach’sα = .79; 1 “nunca”—4 “todo el tiempo”) Never Exposed to at least some poor housing	M = 1.6 (SD = .7), Range 1–4 37.9% (25) 62.1% (41)

### Baseline Clinical Characteristics and Barriers to Care

The US appeared to be the country of transmission for the majority of participants (95%). The average age of diagnosis was 36 years. Over half (68%) were newly diagnosed, and the remaining 32% were returning to care or engaging in care for the first time with an HIV diagnoses of more than 6 months. Half (50%) were referred to the program from acute care settings. Only 9% were virally suppressed at baseline; 35% had documented opportunistic infections (OIs); and 26% concurrent Syphilis. Of those diagnosed within 3 months of consent, 82% were Stage 3 (with CD4 counts < 350 or OIs). Just under one third (30%) reported past or current intimate partner violence concerns; 52% screened positive for depression and 14% screened positive for PTSD. Over half (65%) screened positive for alcohol concerns, 23% for drug concerns, and participants endorsed on average 9 out of 24 barriers to care Table 3.

**Table 3:** Baseline clinical presentation

Variable	Descriptive statistics (n = 66)
Age of diagnosis	M = 35.9 yrs (SD = 9.5), Range 0–59.6
Months since diagnosis	M = 33.1 months (SD = 61.2), Range 0–273.8
Care continuum status Newly diagnosed Not optimally engaged	68.2% (45) 31.87% (21)
Country of transmission US At birth in Mexico Close to time of migration prior to entry into US	95.5% (63) 1.5% (1) 3% (2)
Suppression rate at study entry	9.1% (6)
OIs prior to study entry	34.8% (23)
Syphilis at study entry	25.8% (17)
Stage 3 at study entry (n = 39) < 3 months from Dx with CD4 < 350 and/or OI)	82.1% (32/39)
Acute care referral to intervention	50% (33)
Violence	
I am currently facing violence and, or abuse that threatens my life	−(0)
I have some fears about my safety (emotional security, sexual, physical)	16.7% (11)
I have experienced abuse or domestic violence in the past	13.6% (9)
I have never experienced abuse or domestic violence	69.7% (46)
CES-D (Total score across 20 items, Cronbach's α = .90; 0 “Menos de un día”−3 “5–7 dias”) (n = 66)	M = 18.1 (SD = 7.3), Range 9–38 47.1% (40) screened positive (scores of 16 or higher)
PTSD (Total score across 4 items, Cronbach's α = .80; 0 “Nada”, 1 “Algo”, 2 “Mucho”) ^1^ (n = 65)	M = .86 (SD = 1.25), Range 0–4 15.5% (13) screened positive
Alcohol substance abuse concerns (Cage; 4 Y/N items) (n = 65)	M = 1.2 (SD = 1.1), Range 0–4 58.8% (50) screened positive (at least 1 or higher score)
Drug abuse concerns (Cage; 4 items Y/N) (n = 65)	M = .4 (SD = .9), Range 0–4 23.5% (20) screened positive (at least 1 or higher score)
Barriers to Care (Total score across 24 items, α = .79, 0 “Nada”, 1 “Algo”, 2 “Mucho”, n = 66)	M = 15.7 (SD = 7.9), Range 2–38 Ave 9.9 barriers endorsed (4.4) 1–21 all but 1 endorsed at least 1 barrier

More than half reported barriers to care relating to fatalism, stigma and denial. Specifically, 79% endorsed believing HIV was a death sentence; 73% endorsed not being ready to accept diagnosis; 70% endorsed fearing judgment; 71% endorsed fear of discrimination; 61% endorsed not feeling sick, and 55% endorsed not wanting to be seen receiving care in an HIV clinic Table 4.

**Table 4:** Barriers to care endorsement rate (n = 66*)

Item Description	Nada/not at all	Algo/some what	Mucho/a great deal	Endorsed (somewhat to a great deal)
Pensaba que mi diagnóstico era una sentencia de muerte. (I thought my diagnoses was a death sentence.)	21.2% (14)	16.7% (11)	62.1% (41)	78.8% (52)
No estaba listo para aceptar mi diagnóstico. (I was not ready to accept my diagnosis.)	27.3% (18)	16.7% (11)	56.1% (37)	72.8% (48)
Sentía que podía sufrir discriminación. (I felt I would suffer discrimination.)	28.8% (19)	21.2% (14)	50% (33)	71.2% (47)
Pensaba que podría ser juzgado. (I thought that I would be judged.)	30.3% (20)	21.2% (14)	48.5% (32)	69.7% (46)
No me sentía enfermo. (I did not feel sick, n = 64)	39.1% (25)	17.2% (11)	43.8% (28)	60.9% (39)
No quería que alguien me viera recibiendo atención en una clínica de VIH. (I did not want anyone to see me getting care from an HIV clinic, n = 65)	44.6% (29)	24.6% (16)	30.8% (20)	55.4% (36)
No sabía que existía un tratamiento disponible para mí. (I did not know there was treatment available for me.)	54.5% (36)	25.8% (17)	19.7% (13)	45.5% (30)
No pensaba que el médico hablaría mi idioma. (I did not think the doctor would speak my language.)	54.5% (36)	22.7% (15)	22.7% (15)	45.4% (30)
Tenía miedo del tratamiento. (I was afraid of treatment.)	54.5% (36)	22.7% (15)	22.7% (15)	45.4% (30)
El trayecto a la clínica era muy largo. (The travel time to the clinic was too long.)	56.1% (36)	25.8% (17)	18.2% (12)	43.9% (29)
No quería tomar mi medicina. (I did not want to take medication, n = 65)	60.0% (39)	18.5% (12)	21.5% (14)	40.0% (26)
Pensaba que no podía conseguir atención médica. (I thought I could not obtain medical attention.)	62.1% (41)	15.2% (10)	22.7% (15)	37.9% (25)
No sabía a dónde ir. (I did not know where to go.)	62.1% (41)	19.7% (13)	18.2% (12)	37.9% (25)
No tenía seguro médico. (I did not have medical insurance.)	65.2% (43)	16.7% (11)	18.2% (12)	34.9% (23)
Prefiero utilizar solo la atención de ER o Emergencia y no involucrarme con un proveedor médico regular. (I prefer to utilize care only in the ER and not involve myself with a regular provider.)	68.2% (45)	10.6% (7)	21.2% (14)	31.8% (21)
Pensaba que podía tener problemas legales. (I thought I would have legal problems)	69.7% (46)	12.1% (18)	18.2% (12)	30.3% (20)
No podía pagar por mi atención médica. (I could not pay for my medical care.)	71.2% (47)	13.6% (9)	15.2% (10)	28.8% (19)
Tengo tendencia a no confiar en los proveedores médicos. (I have a tendency to not trust medical providers.)	71.2% (47)	16.7% (11)	12.1% (8)	28.8% (19)
Tenía miedo de lo que mi pareja pudiera hacerme si descubría que estaba recibiendo atención. (I was afraid what my partner would do to me if he or she discovered out I was receiving care.)	71.2% (47)	7.6% (5)	21.2% (14)	28.8% (19)
No contaba con medio de transporte. (I had no transportation.)	72.7% (48)	18.2% (12)	9.1% (6)	27.3% (18)
Nunca antes había visto a un doctor. (I had never before seen a doctor.)	72.7% (48)	12.1% (8)	15.2% (10)	27.3% (18)
No podía obtener permiso en el trabajo. (I could not get time off from work.)	75.8% (50)	19.7% (13)	4.5% (3)	24.2% (16)
He tenido experiencias negativas con proveedores médicos en el pasado y me resisto a buscar atención. (I have had negative experiences with medical providers in the past and am reluctant to seek care.)	77.3% (51)	18.2% (12)	4.5% (3)	22.7% (15)
No contaba con servicio de cuidado infantil. (I had no child care (daycare, n = 48)	89.6% (43)	8.3% (4)	2.1% (1)	10.4% (5)

^*^n = 66 unless otherwise noted

### Characteristics Associated with Barriers to Care and Stage 3 HIV

Correlations were explored between barriers total score and demographic, transnational and clinical variables. Higher barrier reports were significantly associated with higher premigration economic concerns (r = 0.35, p < 0.01), positive PTSD screen (rpb = 0.29, p < 0.05) and positive depression screen (rpb = 0.27, p < 0.05) as well as importantly Stage 3 recruitment (rpb = 0.38, p < 0.05). Upon further exploration, we found a significant association between mental health concerns and Stage 3 HIV (Fisher’s Exact Test, p < 0.05). All of the newly diagnosed participants recruited within 3 months of diagnosis who screened positive for depression also screened positive for PTSD (n = 18) and were identified with Stage 3 HIV. Barriers were normally distributed by Stage 3 HIV (Shapiro–Wilk, ns), and were found to be significantly higher for those identified with Stage 3 HIV (Independent Samples t test (20.25) −3.88, p < 0.01. Those in Stage 3 HIV endorsed 57% more barriers than those not in Stage 3 HIV [M_Stage 3_ = 16.09 (SD = 7.34); M _Not Stage 3_ = 9.00 (SD = 3.42)]. Premigration economic concerns were not significantly associated with Stage 3 recruitment.

## Discussion

This study adds to the literature on HIV linkage to care and care retention associated with migration and adaptation to living in the US, specifically depression, trauma, substance abuse and persistent socioeconomic stress [6, 7, 21]. Participants reported significant pre and postmigration concerns including poverty, social stigma, late entry to care or late stage HIV diagnosis, and concurrent health concerns, including 47% screening positive for depression. Depression and barriers to care were significantly related to Stage 3 HIV.

Individuals in our study endorsed multiple stressors associated with their HIV diagnosis including stigma, shame, and *fatalismo* Table 4. Close to half of our sample reported at least one traumatic incident during their migration to the U.S. and 14% scored positive for PTSD. We did not explore on a deeper level other experiences of trauma, but given our findings, albeit limited in scope, we believe that the likelihood of delayed linkage and/or retention in HIV care is in part connected to the historical and complex trauma associated with migration and adaptation to the US including: social isolation, poverty, violence, discrimination, racism and fear of HIV illness [11, 12, 22, 23]. Further, identifying as a gay man or MSM coupled with the current anti-immigrant socio-political climate adds another layer of discrimination and potentially exacerbates symptoms of trauma, which can lead to maladaptive coping [22, 23] and impact linkage and retention in HIV care [11, 12, 24, 25].

Participants also reported access and logistics barriers. Access to care can be largely contingent on insurance coverage. Insurance coverage, conversely, is largely contingent on employment in sectors that offer worker benefits and protections. The majority of participants in our study were presumed to be undocumented due to their migration details and thus not eligible for Medicaid, despite likely acquiring HIV in the United States. While individuals in Chicago can access HIV care independent of documentation status, the belief that this may be an eligibility requirement to access public health services can further complicate HIV care.

Most of the individuals in our study moved to the U.S. as young adults, in their late teens or early twenties. Adolescence and young adulthood are a universal period of risk taking and sexual exploration. Additionally, multiple studies point to migration as a source of sexual risk taking and vulnerability to HIV for Mexicans [9, 21, 26]. After migration, Mexican immigrants are more likely to have sex with multiple partners, engage in spontaneous sex, condomless sex and meet potential partners at bars [26, 27]. Over half of our sample also endorsed alcohol issues and nearly one quarter reported concerns with other substances, these behaviors collectively, migration and substance use, can elevate HIV risk [7, 8, 21, 28] and appears to be consistent with our findings that the majority of participants in this study appear to have acquired HIV after migration to the United States.

The convergence of all of these issues underscore the need to create programs that are holistic, inclusive and nuanced in scope. While understanding the social characteristics and barriers to care for Mexican immigrants is critical to helping achieve retention in care and viral suppression, understanding these issues alone and creating interventions that address these kinds of barriers are not enough to improve the lived experiences of this Mexican immigrant population.

More attention and advocacy efforts need to be geared towards dismantling the systems of oppression that made these individuals vulnerable to HIV to begin with and that continue to complicate their care retention. A focus on structural barriers, specifically anti-racism work, fighting against patriarchy or in this case machismo, health advocacy and immigration reform can help individuals feel accepted, grounded, safe and further, would support their efforts to get out of the shadows and join the legal workforce, positioning this group to feel more empowered in their day to day lives [29].

Ongoing research is needed to explore and document the social and health ramifications of economic immigration. Our attempt to understand concerns focused more heavily on those related to premigration and journey and then barriers to engaging in HIV care. More research, ideally qualitative, is needed to better understand the entirety of intrapersonal, social and health challenges immigrants and sexual minorities face while navigating U.S. communities. Further, additional research is needed in the design and evaluation of preventive interventions pre and postmigration to bolster mental and sexual health, reduce stigma, reduce transmission of HIV, as well as increase awareness of the importance of routine testing and timely engagement in care. Nearly one quarter of the sample did not identify with any of the sexual orientation options in the survey (heterosexual, *derecho*, straight; homosexual, gay, bisexual, otro). This lack of identification was more likely for those screening positive for depression. Future research could explore meaning, saliency, and emic language relating to sexual orientation.

One significant limitation of this study was the lack of nuanced data that could have been garnered through a qualitative study. This study sheds light on broad social and structural barriers to care for Mexican born men living with HIV in Chicago, but a follow-up study inclusive of in-depth interviews exploring the stories of these individual participants would provide more depth and further our understanding of the barriers to HIV care and retention for this group. Additionally, these findings cannot be generalized to Mexicans as a whole, as the subpopulation in this study was specific to the experiences of Mexican born men living with HIV in Chicago who access care at a safety net public health clinic.

### New Contribution to the Literature

This study uniquely describes the social characteristics and barriers to HIV care and retention for Mexican born/immigrant men living in Chicago. Given that the rates of HIV continue to rise among Latinx communities across the United States and specifically, Mexican men in Chicago, understanding these characteristics and barriers is important to curbing the epidemic among this subpopulation. More prevention and intervention research and action are needed to ameliorate the negative socioeconomic and health ramifications of economic immigration and bolster the mental and sexual health, reduce transmission of HIV, and increase testing, linkage, engagement and retention in HIV care.

## Supplementary Information

Below is the link to the electronic supplementary material.

Supplementary file1 (DOCX 17 KB)
