# Supplementary material for: Challenges and Barriers to HIV Care for Mexican Born Men Living in Chicago
Source: J Immigr Minor Health. 2021 Feb 22:1–9. Online ahead of print. doi: 10.1007/s10903-021-01158-y (PMC7897882; doi:10.1007/s10903-021-01158-y)
Supplement: Supplementary file 1 — Supplementary file1 (DOCX 17 KB) [file 10903_2021_1158_MOESM1_ESM.docx]

**Appendix: Pre and Post Migration Stressors Influenced by Hispanic Stress Inventory**

| **Premigration economic stressors** |
| --- |
| En México, mi familia no tenía dinero para alimentos. (In Mexico, my family did not have money for food.)  En México, me faltaban oportunidades educativas. (In Mexico, I was lacking educational opportunities.)  En México, los miembros de mi familia no podía conseguir una buena atención médica. (In Mexico, members of my family could not obtain good medical attention.)  En México, mi familia luchó para conseguir una vivienda. (In Mexico, my family struggled to obtain housing.)  En México, me mudé de un pequeño pueblo a una ciudad con el fin de cubrir mis necesidades. (In Mexico, I moved from a small town to a city in order to meet my needs.)  En México, yo era responsable del cuidado financiero de otros miembros de mi familia. (In Mexico, I was responsible for caring financially for other family members.) |
| **Premigration social stigma** |
| En México, me sentía como si no encajara en la familia. (In Mexico, I felt like I did not fit in the family.)  En México, me sentía como si no encajara socialmente. (In Mexico, I felt like I did not fit in socially.)  En México, me sentía aislado con muy pocos amigos. (In Mexico, I felt isolated with very few friends.)  En México, la gente se burlaba de mí debido a mi comportamiento, a mi forma de hablar o a la gente con quien me juntaba. (In Mexico, people would make fun of me due to how I acted, the way I talked, or the people I would hang out with.) |
| **Journey trauma** |
| Durante mi viaje a los EUA fui agredido físicamente. (On my journey to the US, I was physically assaulted.)  Durante mi viaje a los EUA fui agredido sexualmente. (On my journey to the US, I was sexually assaulted.)  Durante mi viaje a los EUA me robaron. (On my journey to the US, I was robbed.)  Durante mi viaje a los EUA fui testigo de que otros inmigrantes sufrieron abusos. (On my journey to the US, I witness other immigrants suffer abuse.)  Durante mi viaje a los EUA vi que otros inmigrantes fallecieron. (On my journey to the US, I saw other immigrants die.) |
| **Postmigration housing concerns** |
| Con qué frecuencia ha tenido que aceptar malas condiciones de vivienda? (How often have you had to accept poor housing conditions?)  Con qué frecuencia ha tenido que vivir en una casa en condiciones concurridas? (How often have you had to live in an overcrowded home?) |

1 “nunca/never”, 2 “rara vez/de vez en cuando/rarely or once in a while,” 3 “con frecuencia/frequently”, 4 “todo el tiempo/all of the time”, 8 “prefiero no contestar/prefer not to answer”.
